# Integrated SERS-Microfluidic Sensor Based on Nano-Micro Hierarchical Cactus-like Array Substrates for the Early Diagnosis of Prostate Cancer

**DOI:** 10.3390/bios14120579

**Published:** 2024-11-28

**Authors:** Huakun Jia, Weiyang Meng, Rongke Gao, Yeru Wang, Changbiao Zhan, Yiyue Yu, Haojie Cong, Liandong Yu

**Affiliations:** State Key Laboratory of Chemical Safety, College of Control Science and Engineering, China University of Petroleum (East China), Qingdao 266580, China; jiahuakun@upc.edu.cn (H.J.); z23050033@s.upc.edu.cn (W.M.); yeruwang@s.upc.edu.cn (Y.W.); b22050011@s.upc.edu.cn (C.Z.); s21050053@s.upc.edu.cn (Y.Y.); b23050002@s.upc.edu.cn (H.C.)

**Keywords:** SERS, microfluidic chip, hierarchical interface, exosome, early diagnosis of cancer

## Abstract

The detection and analysis of cancer cell exosomes with high sensitivity and precision are pivotal for the early diagnosis and treatment strategies of prostate cancer. To this end, a microfluidic chip, equipped with a cactus-like array substrate (CAS) based on surface-enhanced Raman spectroscopy (SERS) was designed and fabricated for the detection of exosome concentrations in Lymph Node Carcinoma of the Prostate (LNCaP). Double layers of polystyrene (PS) microspheres were self-assembled onto a polyethylene terephthalate (PET) film to form an ordered cactus-like nanoarray for detection and analysis. By combining EpCAM aptamer-labeled SERS nanoprobes and a CD63 aptamer-labeled CAS, a ‘sandwich’ structure was formed and applied to the microfluidic chips, further enhancing the Raman scattering signal of Raman reporter molecules. The results indicate that the integrated microfluidic sensor exhibits a good linear response within the detection concentration range of 10^5^ particles μL^−1^ to 1 particle μL^−1^. The detection limit of exosomes in cancer cells can reach 1 particle μL^−1^. Therefore, we believed that the CAS integrated microfluidic sensor offers a superior solution for the early diagnosis and therapeutic intervention of prostate cancer.

## 1. Introduction

Prostate cancer ranks among the most prevalent malignancies within the male genitourinary system, with consistently high incidence and mortality rates on a global scale [1,2]. It has shown a persistent upward trend. In 2023, it constituted 29% of all male cancer diagnoses in the United States, underscoring its status as a leading disease in this demographic [3]. Early detection of prostate cancer is paramount for enhancing treatment efficacy. Early diagnosis enables the identification of prostate cancer before extensive metastasis, facilitating more effective therapeutic interventions such as surgical resection and radiation therapy, which are most efficacious when the cancer is localized.

Presently, digital rectal examination (DRE) [4], transrectal ultrasound (TRUS) [5], magnetic resonance imaging (MRI) [6,7,8], and prostate-specific antigen (PSA) testing [9,10] are the primary diagnostic modalities for clinical identification of prostate cancer. DRE and TRUS are straightforward and cost-effective, but they are not sufficient for standalone diagnosis; they must be combined with other examination results, and their ability to detect early-stage prostate cancer is limited. Multi-parametric MRI is recognized as a standard diagnostic method for prostate cancer. However, its implementation necessitates costly equipment, advanced technology, and a high degree of expertise from medical professionals. PSA testing may lead to overdiagnosis and potentially involve unnecessary invasive biopsies and overtreatment. Prostate biopsy can provide reliable detection results, but its invasive nature poses risks such as the potential for local infection and bleeding during the procedure [11]. Therefore, there is an urgent need for a reliable, non-invasive detection method that ensures effective detection while minimizing equipment costs and professional expertise requirements.

Liquid biopsy, an emerging non-invasive diagnostic assay, monitors tumor presence and dynamics by analyzing blood-based biomarkers, including circulating tumor DNA (ctDNA), circulating tumor cells (CTCs), and extracellular vesicles (EVs) [12,13,14]. Several previous reports indicate that liquid biopsy-based approaches could increase the detection rate of early-stage cancer by up to six-fold [15]. Exosomes, one of the detection targets of liquid biopsy, are small vesicles (approximately 40–100 nm in diameter) produced by living cells and enclosed by lipid bilayers. They contain a complex array of RNA and proteins, which can release into the extracellular space to facilitate intercellular communication and modulate cellular functions [16,17]. Detecting and analyzing exosomes derived from human prostate cancer cells enables the acquisition of more detailed and personalized information [18]. Current methods for detecting exosomes encompass transmission electron microscopy (TEM) [19], flow cytometry [20,21], nanoparticle tracking analysis (NTA) [22,23], and resistive pulse sensing [24]. However, the corresponding preparatory steps and substantial cost requirements demand specialized experimental equipment and expertise, highlighting a significant need for expertise and infrastructure. Surface-enhanced Raman spectroscopy (SERS) technology provides a new strategy for the detection of exosomes through its high sensitivity and molecular fingerprint.

SERS is a molecular vibrational spectroscopy technique known for its broad spectral range, low fluorescence background, high sensitivity, minimal water interference, and capability for single-molecule detection [25,26]. These attributes render SERS particularly well-suited for the analysis of biological samples. Consequently, various SERS-based detection strategies were developed to facilitate sensitive exosome analysis. For example, Xie et al. [27] used SERS for label-free spectral analysis of serum exosomes, achieving a prediction accuracy of 100%. This approach could accurately diagnose subtypes of breast cancer. Diao et al. [28] and Shin et al. [29] developed SERS-based exosome analyses to distinguish exosomes originating from various cell lines or derived from the plasma of patients with different cancer types. Although SERS technology offers obvious advantages in the field of biosensing, it still faces challenges in practical applications, such as signal fluctuations, poor reproducibility and stability, the uncontrollable aggregation problems of nanocolloids, and a dependence on specific experimental conditions. Therefore, the preparation of SERS substrates characterized by good stability and superior enhancement efficiency is of paramount importance.

Currently, SERS substrates are categorized into liquid [30,31], solid [32,33,34], and flexible states [35,36,37,38]. The solid substrate is particularly favored for SERS detection owing to its excellent stability and reproducibility. For instance, Zhang et al. [39] developed an Au@Ag nanoparticle/graphene oxide substrate. This substrate offers significant electromagnetic enhancement and a multitude of DNA adsorption sites, thereby enabling a broad detection range and low limit of detection. Li et al. [40] developed a layered SERS substrate, which demonstrated a SERS intensity 3.5 times greater than that of the sandwich immune complex alone. Furthermore, Lin et al. [41] fabricated a nanosheet SERS substrate that achieved an ultra-low limit of detection and enabled single-molecule detection. Although these substrates offer a large specific surface area and electromagnetic field enhancement, they are two-dimensional (2D) planar substrates, and their detection performance needs to be improved compared to three-dimensional (3D) substrates. Because of their intricate geometry, 3D structures are able to generate a greater number of ‘hot spots’ over a larger spatial range, which are crucial for enhancing Raman signals. In addition, the larger specific surface area of 3D substrates allows for increased adsorption of probe molecules, thereby enhancing detection sensitivity and molecular enrichment efficiency. Chen et al. [42] developed a novel 3D surround-enhancing SERS substrate that significantly enhances the SERS signal. This substrate densely covers exosomes with ‘hotspots’ generated by AuNPs, providing sensitive and comprehensive SERS signals. However, this substrate, being a liquid substrate, suffers from stability issues. To address this, Nguyen et al. [43] fabricated a flexible and simple 3D plasmonic-cluster SERS solid substrate, which exhibits significant stability and enhanced SERS intensity, with a limit of detection down to 10^−6^ M for p-nitrophenol molecules. However, the cylindrical design of the 3D cluster substrate, in conjunction with the downward irradiation of the laser, allows for the collection of target signals solely from the top surface. It impedes the collection of signals from the lateral surfaces, thereby potentially compromising the sensitivity of target detection. To overcome this limitation, we developed an orderly nanocone-structured 3D substrate aimed at providing excellent detection performance.

To enhance the feasibility of real-time detection, the integration of a SERS substrate into microfluidic chips, creating a highly sensitive detection platform, represents a burgeoning trend in point-of-care testing (POCT). Usually, a microfluidic chip contains a number of microchannels and micro reaction chambers in a single platform. The microfluid moved within microscale channels and executed precise manipulations, thereby performing essential laboratory functions such as biological interactions, chemical reactions, and environmental analyses [44,45]. Microfluidic chips, characterized by their compact size, cost-effectiveness, and expeditious response times, are of considerable importance in multidisciplinary researches [46]. The integration of SERS with microfluidic chip technology creates a highly sensitive detection technology, which is widely recognized as an important detection method with significant potential for biomedical testing [47].

Herein, we demonstrated a novel integrated microfluidic platform that couples a cactus-like array substrate (CAS) with microfluidics for the high-performance detection of exosomes. The innovation of this work is primarily manifested in two aspects. Firstly, we prepared a novel CAS using 2 μm and 200 nm polystyrene microspheres (PS) with colloid lithography and dry etching techniques. This present CAS generated a considerable number of hotspots, thereby markedly amplifying the Raman signals of the SERS nanoprobes. Secondly, we established a sandwich structure by conjugating exosome surface proteins with both substrates and nanoprobes, thereby successfully capturing exosomes and significantly enhancing their Raman signals. To the best of our knowledge, this is the first application of a SERS-based microfluidic sensor for prostate cancer exosome detection. In summary, we believe that this novel integrated microfluidic platform holds significant potential for the early, rapid, and sensitive diagnosis of prostate cancer.

## 2. Experimental Section

### 2.1. Preparation of a Nucleic Acid Chain Modified CAS

The preparation of the CAS was achieved through three main steps: self-assembly of PS microspheres and nanospheres, inductively coupled plasma (ICP) etching, and electron beam deposition technology. Firstly, a glass slide was cut into small pieces (2 × 2 cm), and after hydrophilic treatment, 500 μL of deionized (DI) water was added dropwise to the surface of the glass pieces. The liquid level could uniformly adhere to the surface of the glass slide, facilitating the self-assembly of colloidal PS microspheres. Then, the PS microsphere solution was prepared by mixing a 2 μm diameter PS microsphere sample with anhydrous ethanol in a ratio of 1:2 and slowly dropping the solution onto a corner of the glass surface. A total of 2 μm of PS nanospheres were observed floating on the liquid surface. Subsequently, an appropriate amount of DI water was added to a beaker and the glass slide was slowly tilted into the beaker to transfer the monolayer of PS microspheres onto the surface of the DI water. Then, a PET film was used to lift the PS microspheres, which were then air-dried to form a single-layer colloidal crystal (SCC). Next, the 200 nm PS nanospheres were mixed with anhydrous ethanol in a ratio of 1:1 and the same method was used to self-assemble the PS nanospheres on the SCC surface, forming a double-layer colloidal crystal (DCC), which was allowed to dry naturally. After that, the prepared DCC was subjected to oxygen plasma etching using an ICP-300 (Institute Of Microelectronics of the Chinese Academy of Sciences, Beijing, China) with an ICP power of 100 W and an RF power of 50 W and an etching time range from 30 to 180 s. Finally, a 30 nm thick gold film was deposited onto the substrate surface using a DZS500 (Sky Technology Development Co. Ltd., Shenyang, China) with a controlled deposition rate of 0.02 nm s^−1^.

The CD63 aptamer was conjugated to the CAS via Au–S bonds. For this process, the CAS was immersed in 200 μL of a solution with a concentration of 1 μmol L^−1^ of the CD63 aptamer and incubated at room temperature for 24 h to facilitate bonding. The thiol groups within the CD63 aptamer formed Au–S bonds with the gold film on the substrate surface. Afterward, the substrate was thoroughly rinsed with deionized water four times and air dried.

### 2.2. Cell Culture and Exosomes Isolation

LNCaP cells were provided by a cell bank (Chinese Academy of Sciences). LNCaP cells were cultured in a complete medium (5% FBS) for a period of three days prior to the exosome isolations. The exosomes released by the cells were isolated using an exosome extraction and purification kit, which was received from UmiBio (Shanghai, China). The purified exosomes were stored in 50–100 μL aliquots and placed in a refrigerator at −80 °C for subsequent exosome detection experiments.

### 2.3. Nucleic Acid Chain-Modified Nanoprobes

The preparation of the colloidal AuNPs has been reported in our previous work [48]. The citrate hydrochloric acid buffer was prepared by a low pH method [49]. Briefly, 0.7353 g of sodium citrate powder was added to 4 mL of DI water, which was then stirred thoroughly to ensure dissolution. Subsequently, hydrochloric acid (HCl) was added to adjust the pH of the solution to 3.0, obtaining the citrate hydrochloric acid buffer. Then, 1 mL of the AuNPs was taken, and 10 μL of a 1 μmol L^−1^ EpCAM aptamer and 2.5 μL of a 2 × 10^−4^ μmol L^−1^ 4-Aminothiophenol (4-ATP) were added, mixing thoroughly. Every 5 min, 10 μL of buffer solution was added, thoroughly mixing the solutions after each addition. This process was repeated once. After 25 min, 70 μL of NaCl was added to the mixture. The solutions were mixed for 30 min, then another 100 μL of NaCl was added. It was mixed thoroughly and left to stand for 1 h. Subsequently, the solutions were centrifuged at 15,000 rpm and 4 °C for 12 min. After removing the supernatant, the nanoprobes were resuspend in 300 μL of 0.5 × PBS.

### 2.4. On-Chip SERS Detection

The SERS measurements reported herein were performed utilizing a Renishaw Invia Raman microscope, operated through Wire 5.5 software. The Raman system was equipped with a helium–neon laser, featuring a wavelength of 633 nm, and a 50× objective lens with a numerical aperture (NA) of 0.75. For each pixel measurement, the experimental parameters were configured with an integration time to 5 s and a laser power set to 1%. The accumulation number for the measurements was set at 5. SERS mapping was performed on a square area (12 μm × 12 μm) of the substrate after immersion in 4-ATP solution for two hours. There were 144 pixels in this area (1 pixel = 1 μm × 1 μm), with each pixel having an exposure time and accumulation number of 1 s and 1. To mitigate the impact of Rayleigh scattering, holographic notch filters were incorporated into the collection path. The laser spot was precisely controlled to a diameter of 1.0 μm on the CAS substrate surface. For each measurement, a random selection of 10 points on the substrate were made, and the mean intensity of the resultant characteristic peaks was determined to be the final statistical outcome.

## 3. Results and Discussion

### 3.1. The Workflow of Exosome Detection on a SERS-Based Microfluidic Chip

**Figure 1:**
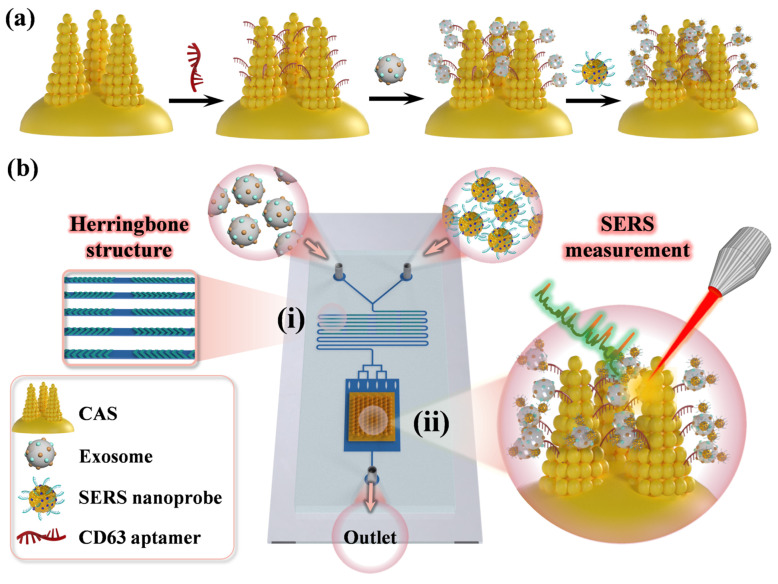
(**a**) Formation steps of the ‘sandwich’ immunocomplexes on CAS. (**b**) Schematic illustration of the SERS-based microfluidic aptamer chip for exosome detection ((i) The mixing channel of each sample; (ii) Rectangular detection chamber embedded with the CAS substrate).

The preparation process of the microfluidic chip was described in detail in the Supplementary Materials. The workflow of detecting human prostate cancer cell exosomes based on a CAS microfluidic chip and the preparation steps of the ‘sandwich’ immunocomplexes on the CAS are shown in Figure 1. Firstly, various concentrations of exosome solution from the prostate cancer cells were injected into one inlet, and 4-ATP labeling EpCAM aptamer-conjugated SERS nanoprobes were injected into the other inlet. The flow rate of each inlet was 1 μL min^−1^. The two solutions flowed into the channel (i) and mixed through the herringbone shallow undulation structures, which disturbed the laminar flow state and reduced the mixing time. The surface proteins of the prostate cancer cell exosomes bound with the EpCAM aptamers on the nanoprobes, fully mixing in the longer S-shaped channel and flowing into a rectangular detection chamber embedded with the CAS substrate, which could be captured by the CD63 aptamer. Finally, a stable formation of ‘sandwich’ immunocomplexes formed on the CAS substrate surface. The Raman signal enhancement on this prepared surface was harnessed to facilitate precise and sensitive detection of the prostate cancer cell exosome concentrations. The formation of the ‘sandwich’ immunocomplexes had good specificity to combine with the large number of SERS hotspots provided by the CAS, enabling the substrate to achieve sensitive and accurate detection. Therefore, our proposed strategy had great potential in the early clinical diagnosis of prostate cancer.

### 3.2. Characterization of CAS

**Figure 2:**
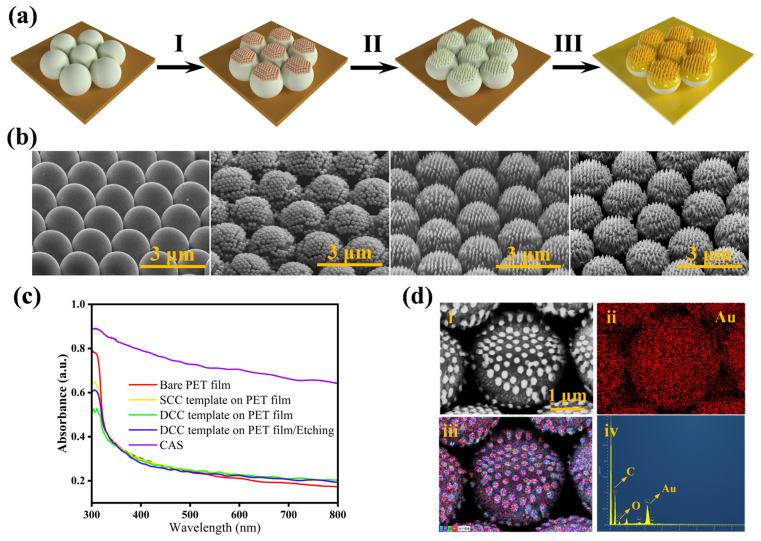
(**a**) Schematic of the fabrication process of the cactus-like array substrate (Step I: Preparation of the DCC template via stacking two SCC templates. Step II: ICP etching of the DCC template. Step III: Evaporation of the gold film). (**b**) SEM images of the CAS fabrication process. (**c**) UV-vis absorption spectra of the substrates with different conditions. (**d**) The SEM images of the CAS (i), EDS elemental maps of Au (ii) and the Au/O/C/N elemental maps on the CAS (iii,iv).

The schematic diagram of each fabrication step of the CAS substrate was shown in Figure 2a. First, two layers of PS spheres were self-assembled on the surface of the PET film, and the substrate surface morphology was constructed by anisotropic etching of 2 μm PS microspheres using inductively coupled plasma (ICP) technology. Finally, a 30 nm layer of gold film was deposited on the substrate surface to complete the substrate fabrication process. The serial changes in surface morphology during substrate fabrication were characterized by scanning electron microscopy (SEM), as shown in Figure 2b. The physical pictures of the different types of substrates under different operations are shown in Figure S1. The SEM images demonstrated that the top layer of the 200 nm PS nanospheres acted as a protective mask during the oxygen plasma etching process, enabling the fabrication of a well-ordered nanocone array. These nanocones, with a height of approximately 100 nm, were formed on the top of the underlying PS spheres. The SEM images depicting the morphological evolution of the CAS surface at various etching stages are presented in Figure S2. The combination of the microspheres and nanocones increased the utilization of laser light by the CAS in the z-axis, and the curved surface formed by the upper surface of the microspheres was able to load a greater number of nanocones in the same area compared to a planar substrate. The UV-Vis absorption spectra, depicted in Figure 2c, illustrate the optical absorption properties of the CASs with distinct morphologies throughout their fabrication process. These results indicate a more than threefold increase in absorbance in the visible light area for the CAS, as opposed to the untreated PET film. This increase underscores the markedly improved capacity of the substrate for light trapping following the gold deposition. Figure 2d(i) shows an SEM image of the top view of the CAS. Furthermore, the energy-dispersive X-ray spectroscopy (EDS) elemental map of the CAS (Figure 2d(ii–iv)) showed that the gold nanoparticles (red dots) were uniformly distributed on the surface of the microspheres, confirming the uniform deposition of gold nanoparticles, which was conducive to achieving a uniform distribution of hot spots.

**Figure 3:**
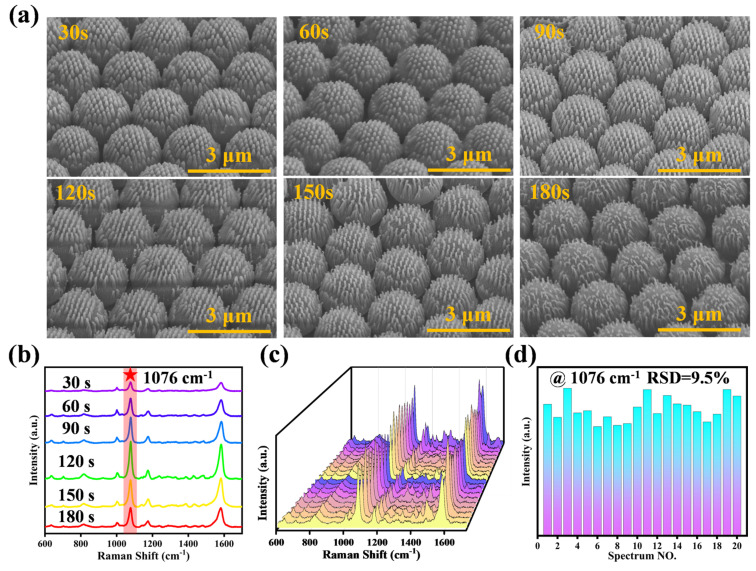
(**a**) SEM images of the DCC template on PET film with different etching times (30, 60, 90, 120, 150, 180 s). (**b**) The SERS spectra of 4-ATP obtained on the CAS with different etching times (30, 60, 90, 120, 150, 180 s). (**c**) 20 SERS spectra randomly measured on the CAS and (**d**) their RSD of the peak intensity @ 1076 cm^−1^.

The evolution of the substrate morphology with increasing etching time was shown in Figure 3a. The 200 nm PS nanospheres gradually shrunk with increasing etching time and eventually disappeared, while the 2 μm PS microspheres formed prominent nanocone structures on their surfaces under the anisotropic etching effect of oxygen plasma. Measurements using image processing software (Image J 1.53e) showed that when the etching time was 120 s, the height of the nanocone on the PS microspheres was 220 ± 30 nm, with an aspect ratio of 1.87:1. As the etching time was further increased, the nanocone structures tended to collapse. The height of the nanocone decreased to 148 ± 20 nm at 180 s, and the aspect ratio reached 1.46:1, indicating that the structures were gradually destroyed.

To delve deeper into the impact of varying etching durations on the SERS performance of the substrate, we meticulously analyzed the distinctive Raman signature of the reporter molecule 4-ATP following the gold film deposition on the substrate. This analysis aimed to ascertain the SERS activity of the substrate under different etching conditions, as shown in Figure 3b. The substrates prepared with different etching times were soaked in a 4-ATP solution with a concentration of 10^−5^ mol L^−1^ for 2 h, and the intensity variation of the characteristic peak at 1076 cm^−1^ was recorded. It indicated that the SERS signal intensities for the characteristic peak of 4-ATP progressively heightened with the etching time and were in a steady rising state before 120 s of labeling. This increase was attributed to the gradually formed nanocone structures on the top of the 2 μm PS microspheres, which increased the surface area of the substrate and promoted the deposition of gold nanoparticles, forming nanogaps smaller than 20 nm. The strong electromagnetic field enhancement effect between adjacent gold nanoparticles generated numerous hot spots, which significantly enhanced the SERS signal. However, when the etching time exceeded 120 s, the SERS intensities showed a decreasing trend. This decrease may be caused by the gradual destruction of the nanocone structures during continuous etching, which affected the deposition sites of the gold nanoparticles and the surface plasmon resonance effect, thereby weakening the signal enhancement effect on the substrate surface. The results showed that the nanocone structures with 120 s etching retained optimal morphological characteristics and exhibited the best SERS response. In addition, to evaluate the signal uniformity of the prepared substrate, the substrate was soaked in a 4-ATP solution for 2 h, and Raman signal tests were performed at 20 randomly selected points on the substrate’s surface. The exposure time and accumulation number for each point were set to 1. The laser scanning range, spanning from 600 cm^−1^ to 1700 cm^−1^, encompassed the characteristic peaks of 4-ATP. The intensity of the 1076 cm^−1^ characteristic peak of 4-ATP, which is indicative of its presence, is illustrated in Figure S3. Figure 3c shows the SERS spectra of the 20 randomly selected points, while the intensity of the characteristic peak at 1076 cm^−1^ is shown in Figure 3d, with a relative standard deviation (RSD) of 9.5% and demonstrating a comparable reproducibility. Furthermore, a SERS mapping image was obtained from the Raman intensity values of 12 × 12 points, which also displayed high uniformity (Figure S4). Based on these results, we selected an etching time of 120 s and performed next SERS detection of exosomes under this condition.

**Figure 4:**
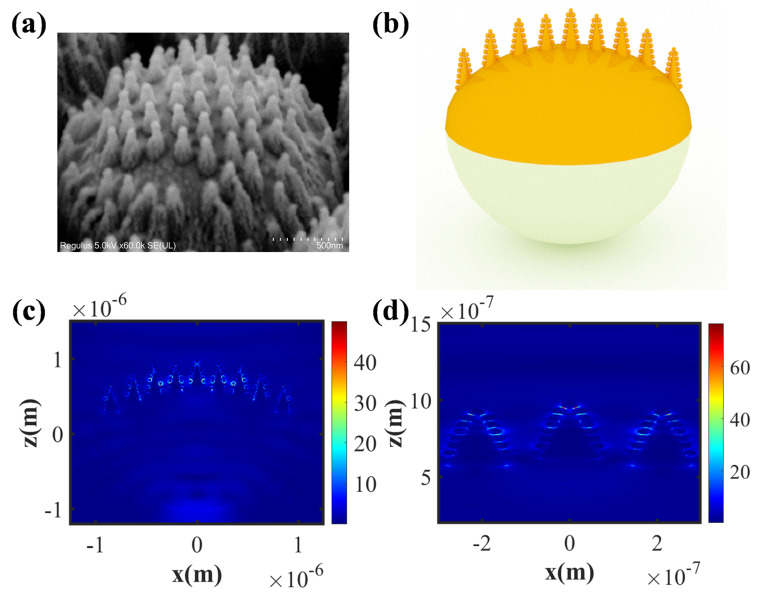
Local FDTD simulation of the CAS. (**a**,**b**) A representative SEM image and schematic of the CAS. (**c**,**d**) FDTD simulation of the electric field distribution on the CAS.

To further elucidate the mechanism of hot spot generation on the substrate surface and its distribution, we performed a simulation analysis of the normalized electric field on the substrate surface using the finite-difference time-domain (FDTD) method. Figure 4a shows a representative SEM image of the CAS with an etching time of 120 s. Based on this image, the substrate was modeled using the 3ds Max (2023) software, as shown in Figure 4b. Figure 4c,d illustrate the electric field intensity distribution (|E/E_0_|) on the substrate surface when the laser wavelength was 633 nm. The simulation results showed that after the gold film deposition, the incident light excited the surface plasmon resonance effect on the gold nanoparticles, forming distributed hot spot regions on the nanocone structures and the gaps between adjacent gold nanoparticles. The emergence of these hot spots substantiates the SERS enhancement effect achieved by the CAS, underscoring its potential for sensitive detection applications.

### 3.3. Feasibility of Exosomes Detection on CAS

**Figure 5:**
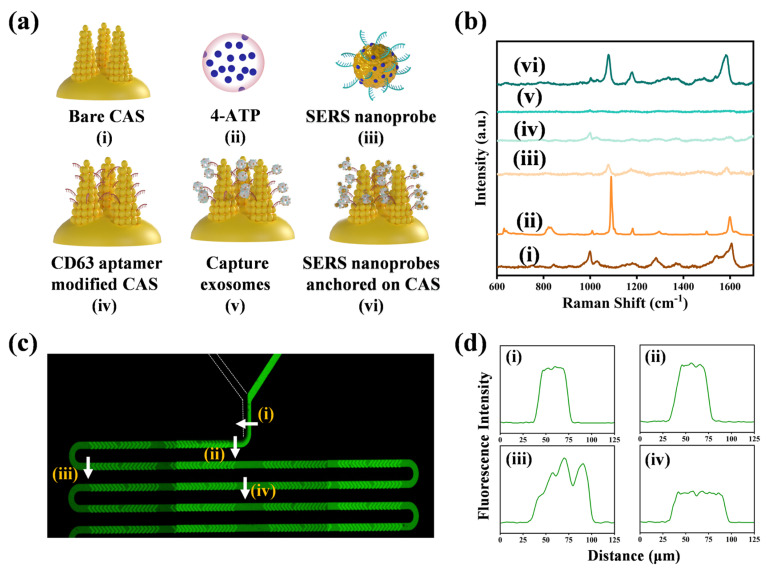
(**a**,**b**) Feasibility test of exosome detection via sandwich immunocomplexes. (**c**) Fluorescent image of the entire microfluidic channels. The white arrows indicated the line profile locations. (**d**) Corresponding fluorescence intensity profiles at four selected locations.

Figure 5a(i–vi) illustrates the process of exosome detection, with the corresponding SERS spectra shown in Figure 5b. It could be clearly observed that the SERS spectrum in Figure 5b(vi) exhibited distinct characteristic peaks of 4-ATP, which were attributed by the gold nanoparticles from SERS nanoprobes and CAS together. Figure 5b(iii) illustrates the SERS characteristic peak of the nanoprobes, and the photograph of the salt aging experiments between the nanoprobes and AuNPs is shown in Figure S5. In addition, the intensity of the characteristic SERS peaks in Figure 5b(vi) are significantly higher than that in Figure 5b(iii), indicating that the substrate further enhanced the SERS signal of the nanoprobes. Specifically, the proteins present on the exosome surface engage with the EpCAM aptamer-labeled nanoprobes, which then interact with the CD63 aptamer-labeled nanoprobes immobilized on the CAS. This interaction culminates in the formation of a comprehensive sandwich immunocomplex. Due to the SERS signal enhancement effect of the substrate, this method enabled high-sensitivity detection of the exosomes, making it a promising approach for early diagnosis of prostate cancer.

### 3.4. The Mixing Efficiency of the Channel

The efficacy and swiftness of the exosomes and SERS nanoprobes mixing within microfluidic chips directly affected the binding efficacy and the ensuing SERS measurement. The physical picture of the microfluidic chip is shown in Figure S6. The 4-ATP labeling EpCAM aptamer-conjugated SERS nanoprobes combined with surface proteins of the exosomes in the herringbone bas-relief channel. After flowing into the CAS-integrated microchamber, they were captured by the CD63 aptamer and modified on the substrate surface to form sandwich immunocomplexes, which directly related to the final SERS measurement results of the exosomes. Therefore, in order to verify the mixing performance of the herringbone bas-relief channel in the microfluidic chip, four specific channel positions in the chip were selected for fluorescence intensity measurement. A 10^−5^ M solution of fluorescein sodium salt (FSS) and deionized water were injected into the two entrances of the microfluidic chip. Subsequently, an inverted fluorescence microscope (Ti2-U) was utilized to acquire the fluorescence spectra at the four distinct locations, as depicted in Figure 5c,d. FSS exhibited green fluorescence under blue laser excitation of fluorescence microscope, while deionized water remained unchanged. The two solutions were in laminar contact with a clear boundary at positions (i) and (ii). After passing through the shallow relief channel of the herringbone shape, the two solutions were fully folded and mixed, showing an unstable state of gradually mixing at position (iii). Continuing to flow through several zigzag channels, the sample was fully mixed at position (iv), and the fluorescence intensity tended to stabilize. Therefore, it indicated that the exosomes and SERS nanoprobes can be fully mixed in microfluidic channel, ultimately flowing into rectangular measurement chamber embedded with the CAS.

### 3.5. Performance of the Exosome Detection Using SERS-Based Microfluidics

**Figure 6:**
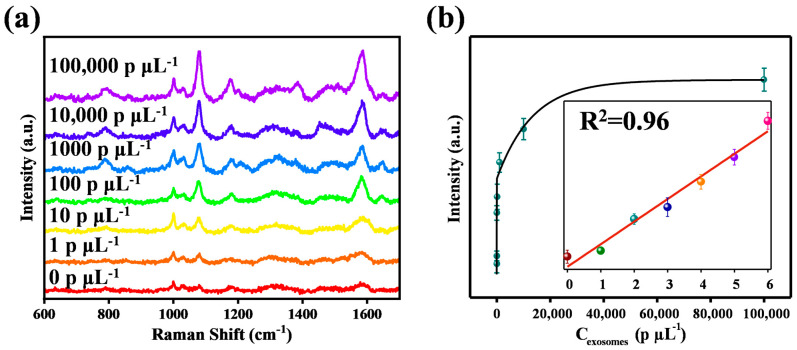
(**a**) The SERS intensity variations with the increase in exosome concentrations. (**b**) Corresponding calibration curves for the exosomes. Inset was the plot of the SERS intensity at 1076 cm^−1^ versus the logarithm of the exosome concentrations. The error bars represent the standard deviations of five individual substrates.

The concentration of the exosomes derived from the prostate cancer cells was detected on a microfluidic chip based on a CAS, and the SERS measurement results are shown in Figure 6. Two inlets on the chip were respectively filled with the exosomes and the AuNPs labeled with 4-ATP and the EpCAM aptamer, with a ratio of 1:1. After the solution was fully mixed and filled the entire rectangular detection chamber, the reaction was carried out at room temperature. The EpCAM protein and CD63 protein on the surface of the exosomes bind to the SERS nanoprobes and CAS, respectively, forming a sandwich structure. The signal intensity value of the 4-ATP at 1076 cm^−1^ was then used as a criterion for determining the concentration of exosomes. Upon irradiation by the incident laser focused on the CAS surface, the nanoprobes and the nanocone array undergo intense coupling resonance. This interaction significantly amplifies the signal intensity emanating from the nanoprobes, thereby enhancing the detection sensitivity. Each SERS spectrum was obtained by randomly selecting 10 points and averaging them. The final results were shown in Figure 6a. The peak at 1076 cm^−1^ was obvious in the SERS spectrum and was selected as a reference to analyze the correlation between exosome concentration and SERS response. Moreover, as shown in Figure 6b, SERS intensities versus the logarithm of exosome concentrations had a good linear correlation within the concentration range of 1 particles μL^−1^–10^5^ particles μL^−1^, spanning over five orders of magnitude. The fitting equation, y = 187.47 × logC − 22.2, had a correlation coefficient (R^2^) of 0.96, where y represents the SERS intensity at 1076 cm^−1^ and C is the exosomes concentration, respectively. The detection limit of the exosomes in prostate cancer cells can reach 1 particle μL^−1^. As shown in Table S1, the proposed exosome identification method exhibited good performance compared to other methods. To verify the potential value in clinical applications of the proposed microfluidic SERS sensor, whole blood samples from a PC patient and healthy volunteer, who consented to participate in this work and signed informed consent, were received from The Affiliated Hospital of Qingdao University. The samples were stored in EDTA-K2 tubes and used within 1 day after collection. In this experiment, SERS measurements were performed on the serum from two blood samples using our microfluidic sensor. As shown in Figure S7, the signal intensity of the peak at 1076 cm^−1^ detected from the cancer patient sample is higher than that from the healthy volunteer sample, indicating that our SERS-microfluidic sensor has the potential value in clinical applications. Thus, we believed that the proposed detection strategy would be possible to offering a promising route toward screening test and early diagnostic of cancer diseases.

## 4. Conclusions

In summary, we reported a SERS-based microfluidic sensor, integrated with the newly developed CASs and designed for the expedited and sensitive identification of exosomes originating from human prostate cancer cells. The CASs were engineered to feature uniform nanocone structures on the surface of 2 μm PS microspheres, which significantly increases the nanoscale surface area. This architectural modification offers an excellent platform for the subsequent functionalization of the aptamer and the generation of SERS hot spots. The nanoscale gaps, resulting from the deposition of the nanocone and AuNPs, display distinctive light trapping properties and enhanced SERS activity. Furthermore, we conjugated CD63 and EpCAM aptamers to the substrate and nanoprobes, respectively, to form sandwich immunocomplexes by interacting with their corresponding proteins on the exosome surface. The CASs exhibited strong SERS signals, facilitating the sensitive detection of the exosomes in prostate cancer cells down to a detection limit of 1 particle μL^−1^. We anticipated that this CAS-integrated microfluidic sensor would prove to be a valuable tool for the early diagnosis of prostate cancer.

## Data Availability

The data presented in this study are available on request from the corresponding author.

## Supplementary Materials

The following supporting information can be downloaded at: https://www.mdpi.com/article/10.3390/bios14120579/s1, Figure S1: Photographs of each stage of CAS fabrication process; Figure S2: SEM images of the CAS with different etching time (30, 60, 90, 120, 150, 180 s); Figure S3: The intensity profile of 4-ATP at 1076 cm^−1^ on CAS with different etching time (30, 60, 90, 120, 150, 180 s); Figure S4: SERS mapping results obtained based on the 1076 cm^−1^ peak. The concentration of 4-ATP is 10^−4^ mol L^−1^, the scale bar is 5 µm; Figure S5: The photograph of salt aging experiments; Figure S6: The photograph of the entire microfluidic chip filled with red ink; Figure S7: Raman spectra obtained by detecting clinical samples using SERS-microfluidic platform; Table S1: Comparison of the performance of various methods for exosome recognition [50,51,52,53,54,55,56,57,58,59,60,61,62,63].
