# Supplementary material for: Integrated SERS-Microfluidic Sensor Based on Nano-Micro Hierarchical Cactus-like Array Substrates for the Early Diagnosis of Prostate Cancer
Source: Biosensors (Basel). 2024 Nov 28;14(12):579. doi: 10.3390/bios14120579 (PMC11674406; doi:10.3390/bios14120579)
Supplement: Supplementary file 1 [file biosensors-14-00579-s001.zip › biosensors-3304851-supplementary.pdf]

Supplementary Material

Integrated SERS-microfluidic sensor based on nano-micro hierarchical cactus-like array substrates for the early diagnosis of prostate cancer

Huakun Jia, Weiyang Meng, Rongke Gao,* Yeru Wang, Changbiao Zhan, Yiyue Yu, Haojie Cong, Liandong Yu*

College of Control Science and Engineering, State Key Laboratory of Chemical Safety, China University of Petroleum (East China), Qingdao 266580, China.

*** Address for correspondence:**

Rongke Gao

Telephone: +86-13023091659; E-mail addresses: rkgao@upc.edu.cn

Liandong Yu

E-mail addresses: liandongyu@upc.edu.cn

List of Contents

1. Experimental Section

2. Figures

3. Table

1. Experimental Section

1.1. *Reagent and instruments*

Polystyrene spheres (2 μm, 5%, w/v and 200 nm, 5%, w/v) were obtained from Huge Biotechnology (Shanghai, China). Polyethylene terephthalate (PET) films with a thickness of 0.15 mm were purchased from Feixia Rubber & Plastic (Shanghai, China). Absolute ethanol (⩾99.8%) was purchased from Sinopharm Chemical Reagent Co., Ltd. (Shanghai, China). 4-Aminothiophenol (4-ATP, 97 %) and triton-X-100 (TX100, 10 % in water) were obtained from Sigma-Aldrich (St. Louis, MO, USA). Sodium chloride (NaCl) was obtained from Aladdin (Shanghai, China). PBS buffer solution was purchased from BBI Life Sciences Co., Ltd. (Shanghai, China). Poly(dimethylsiloxane) (PDMS, Sylgard 184) and the curing agent were purchased from Dow Corning. Exosome extraction and purification kits were received from umibio (Shanghai, China). EpCAM nucleic acid aptamers and CD63 nucleic acid aptamers were purified and synthesized by Sangon Biotech Co., Ltd. (Shanghai, China).

Deionized water (18.2 M Ω) was prepared using a water purification system (SMARTPLUS-N, Heal Force Bio-MediTech Holdings Ltd., China). A plasma cleaning system (PDC-32G-2, Harrick Plasma, USA) was used to perform hydrophilic treatment on the device surface. An inductively coupled plasma (ICP) etching system (ICP-300, Chinese Academy of Sciences) was used to etch the substrate and form a nanocone structure. A 30 nm gold film was deposited onto a substrate using an electron beam deposition system (DZS500, Sky Technology Development Co., Ltd., China). SEM images of the substrate were obtained using a scanning electron microscope (Regulus

8100, Hitachi Ltd., Japan). Raman signals were measured and Raman spectra were obtained using a Raman microscopy imaging system equipped with a 633 nm laser (Renishaw inVia Reflex, Renishaw Ltd., UK).

1.2 ***The integrated microfluidic chip fabrication***

Microfluidic chips were developed for the detection of exosomes derived from prostate cancer cells. It was designed with three distinct sections: a solution injection segment, a mixing reaction segment, and a SERS detection segment. The chip was devised two injection ports for the introduction of the exosomes from prostate cancer cells and gold nanoparticles functionalized with EpCAM nucleic acid sequences. Additionally, the chip includes a mixing channel with a distinctive zigzag structure, shallow undulating structure atop the channel, as shown in Figure 1(i). This design enhances fluid motion at low Reynolds numbers, thereby promoting efficient reaction and thorough mixing of the two liquids within the microfluidic channel. The third segment is a rectangular SERS detection chamber, which measures 8.5 mm in length and 6.2 mm in width, as illustrated in Figure 1(ii). This chamber contains an embedded CAS, which is designed to capture the exosomes derived from prostate cancer cells, form a sandwich structure with nanotags and detect their SERS signals.

The fabrication of the microfluidic chip employed standard ultraviolet (UV) lithography and polymer rapid prototyping methods. Initially, the first layer of SU-8 3035 negative photoresist (MicroChem, USA) was spun at 1300 rpm to achieve a thickness of 70 μm and exposed using a patterned photomask on a mask aligner (MDA-

400LJ, MIDAS, Korea). Subsequently, the second photoresist layer was spun at 3500 rpm, a 30 μm herringbone structure was created. The main channel width of the microfluidic chip, excluding the rectangular detection chamber, was measured to be 200 μm. The PDMS prepolymer was then mixed with a curing agent at a mass ratio of 10:1. To eliminate air bubbles from the mixture, a vacuum degassing process was applied. A stainless-steel block, tailored to match the SERS substrate, was positioned to form a rectangular chamber. The PDMS mixture was then poured into the mold, degassed to remove residual bubbles, and subsequently cured in an oven at 70°C for two hours. Afterward, the PDMS layer was removed, and the stainless-steel block was detached. Both the glass slide and the PDMS layer underwent oxygen plasma treatment to enhance adhesion. The CAS substrate was swiftly embedded into the rectangular cavity of the PDMS layer and firmly bonded to the glass slide.

2. Figures

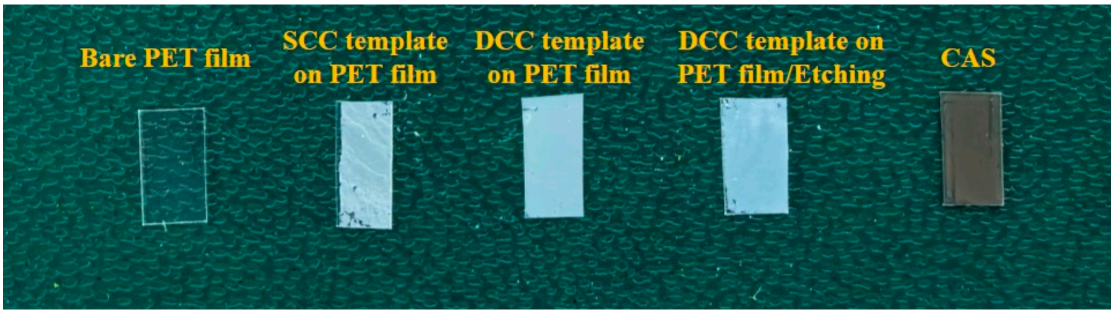

Figure S1. Photographs of each stage of CAS fabrication process.

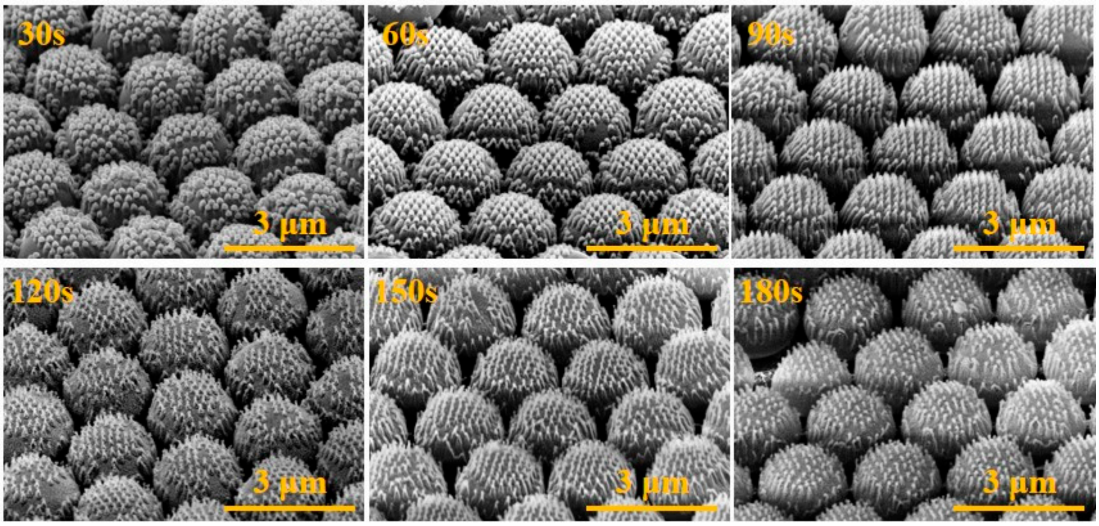

Figure S2. SEM images of the CAS with different etching time (30, 60, 90, 120, 150, 180 seconds).

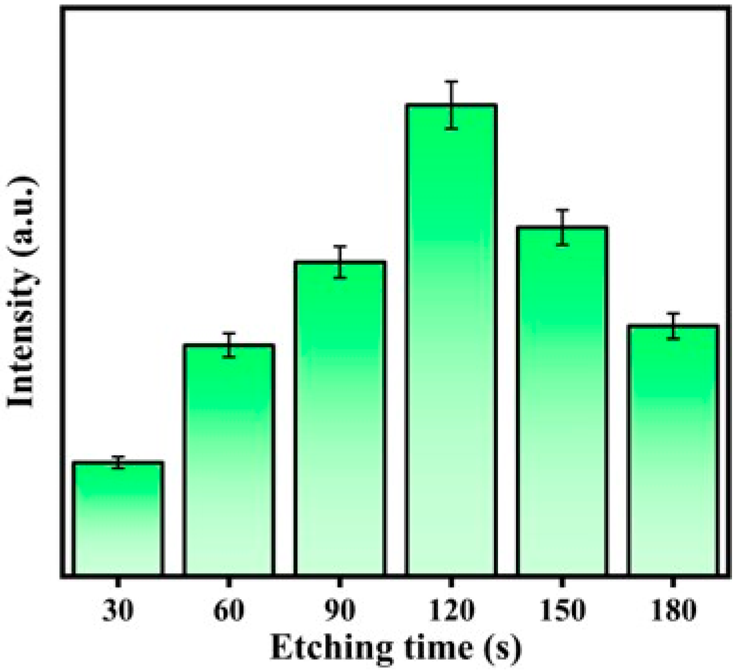

Figure S3. The intensity profile of 4-ATP at 1076 cm^{-1} on CAS with different etching time (30, 60, 90, 120, 150, 180 seconds).

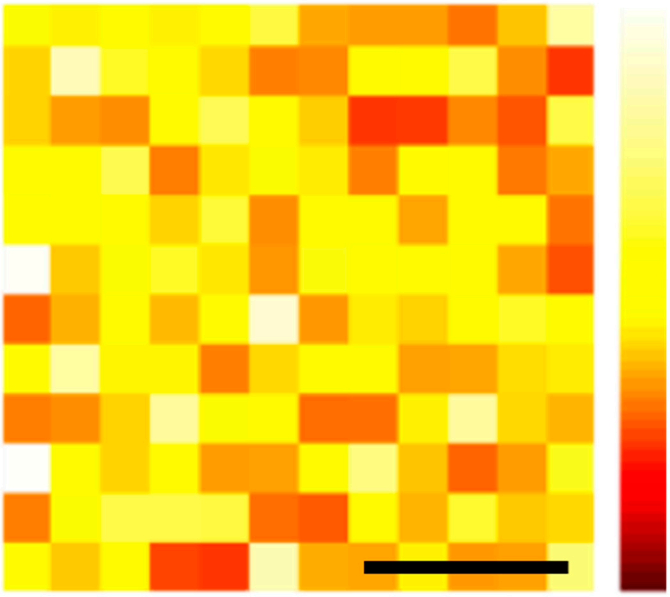

Figure S4. SERS mapping results obtained based on the 1076 cm^{-1} peak. The concentration of 4-ATP is 10^{-4} mol L^{-1}, the scale bar is 5 μm.

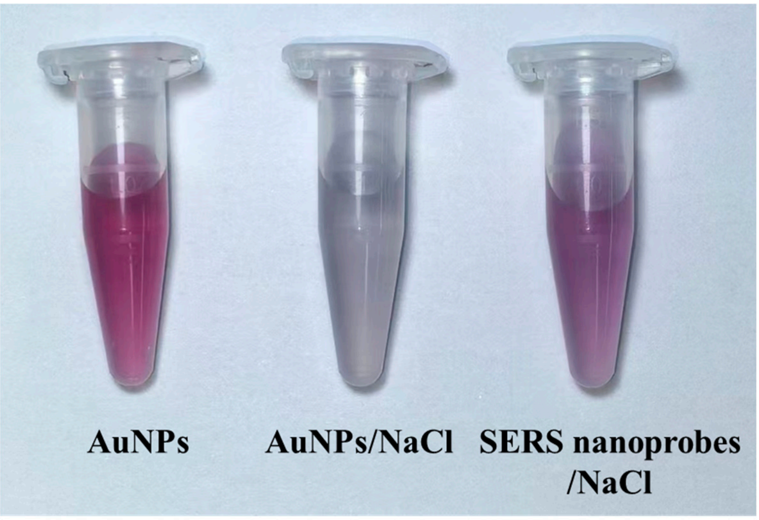

Figure S5. The photograph of salt aging experiments.

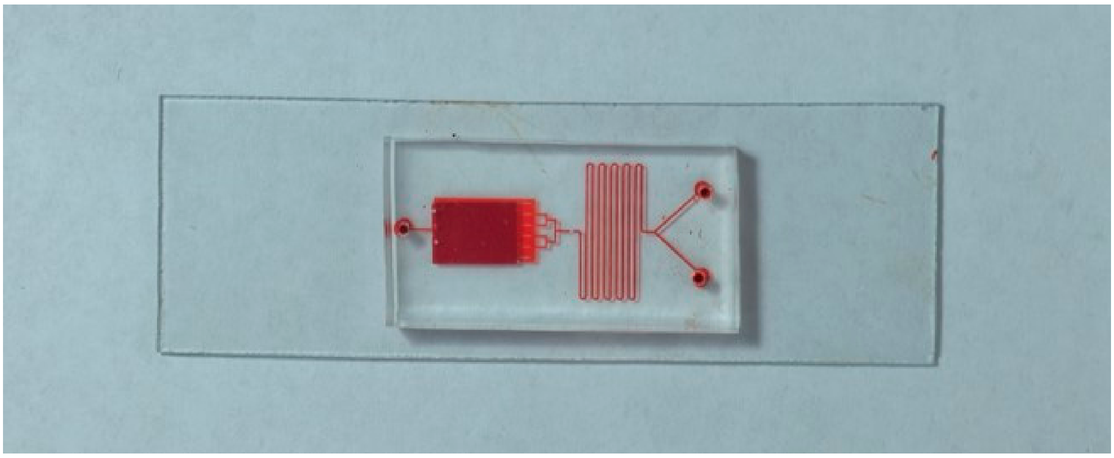

Figure S6. The photograph of the entire microfluidic chip filled with red ink.

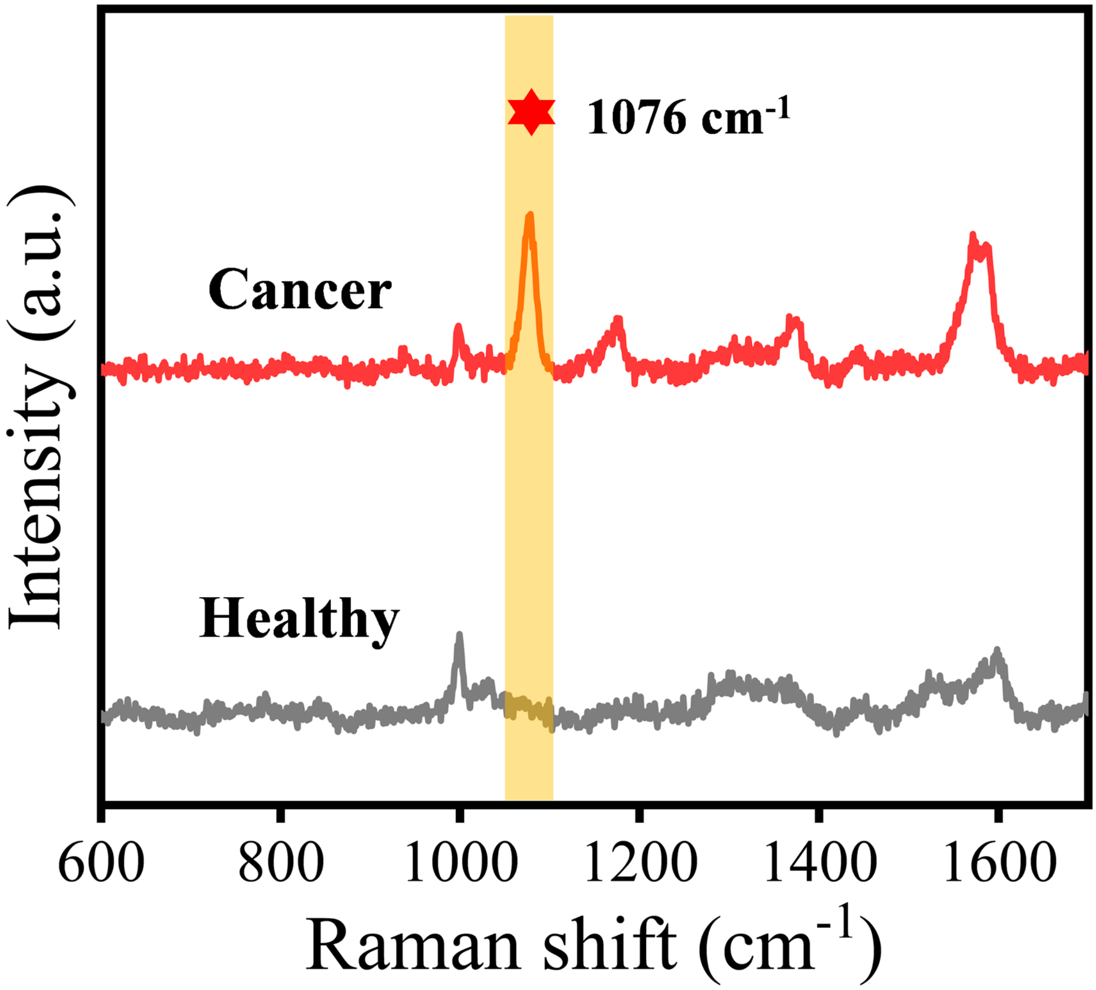

Figure S7. SERS spectra obtained by detecting two clinical samples using SERS-microfluidic platform.

3. Table

Table S1. Comparison of the performance of various methods for exosome recognition.

Method	Strategy	Target	Analysis times	Dynamic range (p/μl)	LOD (p/μl)	Ref
FL	terminal deoxynucleotidyl transferase-mediated signal amplification	CD63	1.5 h	3.6×10^{2} - 7.19×10^{6}	3.6×10^{2}	50
	CHA integrates agarose-based microfluidic chip	CD63	2.5 h	$0\text{-}10^{5}$	10^{3}	51
	centrifugal microfluidic disc system combined with functionalized membranes	PD-L1	8 min	1×10^{1}-1×10^{5}	1.58×10^{2}	52
CA	spherical nucleic acid-induced HCR	CD63	90 min	1×10^{2}-1×10^{3}	50	53
	nano enzyme-functionalized flower-like DNA structures via RCA	EpCAM	120 min	2×10^{3}-4×10^{4}	1.027×10^{3}	54
EC	click chemistry and HCR	CD63	3 h	1.12×10^{2} - 1.12×10^{8}	96	55
	microfluidic integrate biosensors	EpCAM	<1 h	1×10^{2} - 1×10^{9}	17	56
	multidirectional HCR	EpCAM	100 min (HCR 60 min)	5×10^{2}-1×10^{5}	285	57
	Dual signal amplification	EGFR	>2 h	5×10^{1} - 5×10^{6}	0.149	58
	DNA-functionalized covalent organic framework capsules	CD63	>7 h	2.5×10^{2} - 2.5×10^{7}	87	59
SERS	SERS Gold Nanorods and a miniaturized device	HER2, EpCAM	2 h	5×10^{1} - 5×10^{5}	2×10^{3}	60
	acoustofluidics-assisted biosensors	CD63	10 min	10^{2} - 10^{8}	10^{2}	61
	plasmonic nanobowtiefluidic device	EVs	N/A	10^{2}-10^{5}	1.32×10^{2}	62
	a microfluidic-SERS platform with RCA	CD63	N/A	1 pM-1 μM	1 pM	63
	microfluidic-SERS platform with CAS	CD63& EpCAM	60 min	$0\text{-}10^{5}$	1	This work

Ref: references; FL: fluorescence Assay; CA: colorimetric assay; EC: electrochemical; CAS: cactus-like array substrates.
